# Surveillance of Summer Mortality and Preparedness to Reduce the Health Impact of Heat Waves in Italy

**DOI:** 10.3390/ijerph7052256

**Published:** 2010-05-06

**Authors:** Paola Michelozzi, Francesca K. de’ Donato, Anna Maria Bargagli, Daniela D’Ippoliti, Manuela De Sario, Claudia Marino, Patrizia Schifano, Giovanna Cappai, Michela Leone, Ursula Kirchmayer, Martina Ventura, Marta di Gennaro, Marco Leonardi, Fabrizio Oleari, Annamaria De Martino, Carlo A. Perucci

**Affiliations:** 1 Lazio Region Department of Epidemiology, Via di Santa Costanza, 53-00198 Rome, Italy; E-Mails: dedonato@asplazio.it (F.K.D.); bargagli@asplazio.it (A.M.B.); dippoliti@asplazio.it (D.D.); desario@asplazio.it (M.D.S.); marino@asplazio.it (C.M.); schifano@asplazio.it (P.S.); cappai@asplazio.it (G.C.); leone@asplazio.it (M.L.); kirchmayer@asplazio.it (U.K.); ventura@asplazio.it (M.V.); perucci@asplazio.it (C.A.P.); 2 Department of Civil Protection, Via Ulpiano, 11-00193 Rome, Italy; E-Mails: marta.digennaro@protezionecivile.it (M.D.G.); marco.leonardi@protezonecivile.it (M.L.); 3 Ministry of Health, Lungotevere Ripa, 1-00153 Rome, Italy; E-Mails: f.oleari@sanita.it (F.O.); a.demartino@sanita.it (A.D.M.)

**Keywords:** heat prevention plan, heat waves, mortality, HHWWS

## Abstract

Since 2004, the Italian Department for Civil Protection and the Ministry of Health have implemented a national program for the prevention of heat-health effects during summer, which to-date includes 34 major cities and 93% of the residents aged 65 years and over. The Italian program represents an important example of an integrated approach to prevent the impact of heat on health, comprising Heat Health Watch Warning Systems, a mortality surveillance system and prevention activities targeted to susceptible subgroups. City-specific warning systems are based on the relationship between temperature and mortality and serve as basis for the modulation of prevention measures. Local prevention activities, based on the guidelines defined by the Ministry of Health, are constructed around the infrastructures and services available. A key component of the prevention program is the identification of susceptible individuals and the active surveillance by General Practitioners, medical personnel and social workers. The mortality surveillance system enables the timely estimation of the impact of heat, and heat waves, on mortality during summer as well as to the evaluation of warning systems and prevention programs. Considering future predictions of climate change, the implementation of effective prevention programs, targeted to high risk subjects, become a priority in the public health agenda.

## 1. Introduction

A recent European study has evaluated the effect of high temperatures on health in European cities, showing the highest impact on the population residing in Mediterranean areas [1–3]. The vulnerability to heat is related to the populations’ susceptibility, extreme weather event characteristics and adaptation measures and prevention actions put in place. The adverse health effects of heat waves are partially preventable, and after the 2003 heat wave, many European countries have devoted considerable resources into reducing the impact of extreme temperatures on health. Warning systems and a number of specific public health interventions have been widely introduced, although the actual level of implementation of different response measures is heterogeneous [4] and evidence of effectiveness of specific preventive activities is still lacking.

Italy is one of the European countries mostly affected by heat waves, and every summer, there is a quantifiable burden of mortality and morbidity associated with the onset of heat waves [5,6]. Recent climate change scenarios have confirmed predictions of an increase in the frequency and intensity of extreme events in southern and central Europe [7,8], and in the future the impact of heat waves on health could have greater public health significance.

Since 2004, the Department of Civil Protection and the Ministry of Health, have implemented a national program for the prevention of heat health effects focused on the elderly and including all regional capitals and cities with more than 200,000 inhabitants. Starting from the largest urban areas, the program was gradually extended to all regions, and to date, has reached national coverage, to include 34 major cities and 93% of the residents aged 65 years and over.

The Lazio Region Department of Epidemiology, identified as the National Coordination Center, centrally manages the city-specific warning systems, whereas at the local level the designated center in charge (Local Civil Protection, Municipality, and Local Health Authority) is responsible for the information network and coordination of the local prevention plan. A National mortality surveillance system was also activated for a “real time” monitoring of summer mortality; the system allows for the assessment of the impact of heat on daily mortality and the detection of increments in mortality associated with heat waves.

The main components of the national program for the prevention of heat-health effects are the:

- city-specific Heat Health Watch Warning Systems (HHWWS);
- local network for the distribution of the warning bulletin;
- national prevention guidelines;
- local registries of at-risk subgroups of the population;
- a rapid “real time” mortality surveillance system;
- evaluation of warning systems and prevention programs introduced.

This article gives an overview of the Italian national program for the prevention of heat health effects, describing the methodologies adopted for the implementation of warning systems, the mortality surveillance system and prevention plans.

## 2. Heat Health Watch Warning Systems (HHWWS)

The introduction of HHWWS, capable of predicting extreme weather conditions and their impact on health, are a key component in the planning and management of heat-health prevention programs [4]. The Italian HHWWS models are based on a retrospective analysis of mortality data and weather variables to identify conditions associated with a significant increase in daily mortality [9].

In the largest urban areas, and where more extensive mortality and meteorological datasets are available, HHWWS are based on the air mass-based approach developed by Kalkstein *et al.* [10,11], which considers weather conditions as a whole. A series of meteorological variables (air temperature, dew point temperature, atmospheric pressure, wind speed and direction) are categorized into air masses using the spatial synoptic approach. For each city, oppressive air masses are identified as those associated with an excess in mortality. The latter is calculated as the difference between observed and daily baseline values. In the Italian cities, two oppressive air masses have been identified as high risk for health: Moist Tropical plus (MT+), characterized by hot humid conditions, observed mainly in the northern cities, and Dry Tropical (DT), characterized by clear skies and hot, dry conditions, observed mainly in the southern and central cities.

For each city, algorithms for the prediction of excess mortality in each oppressive air mass are defined, and the model includes both minimum and maximum temperatures, time of season, degree hours (sum of degrees centigrade above 20 °C for each of the four temperature values considered during the day) and the days in sequence of oppressive air masses [12]. A warning is issued when an oppressive air mass with a significant excess in mortality is predicted.

The estimated impact of each air masses on mortality differs among cities, with the mean percent increase in daily mortality for MT+ ranging from +15% to +46% and for DT from 7% to 20% [16].

For all cities a second model was developed, based on maximum apparent temperature (Tappmax) (Table 1). The latter is a discomfort index based on air and dew point temperature, calculated using the following formula [10]:

$$T_{\text{app}}=-2.653+0.994(T_{\text{air}})+0.0153{\left(T_{\text{dewpt}}\right)}^{2}$$

In this second approach, the relationship between mortality and Tappmax is investigated through a Poisson regression model for each city. The explicative variables included in the model are: holidays, month (May–August), the interaction between Tappmax and month, and the number of consecutive hot days with Tappmax above the threshold. For each value of Tappmax, the model estimates the associated increase in daily mortality and, on the basis of these results, defines monthly thresholds. A progressive increase in threshold levels was observed, thus accounting for the change in adaptation processes throughout the summer season (Table 1). This model implies a graded classification of risk defined as low (<20% excess in mortality) and high (≥20% excess in mortality).

In the cities where the air mass based model is used, the Tappmax model that predicts at risk conditions (excess mortality) in a continuous way ensures that pre-warning conditions are also identified.

**Figure 1.**
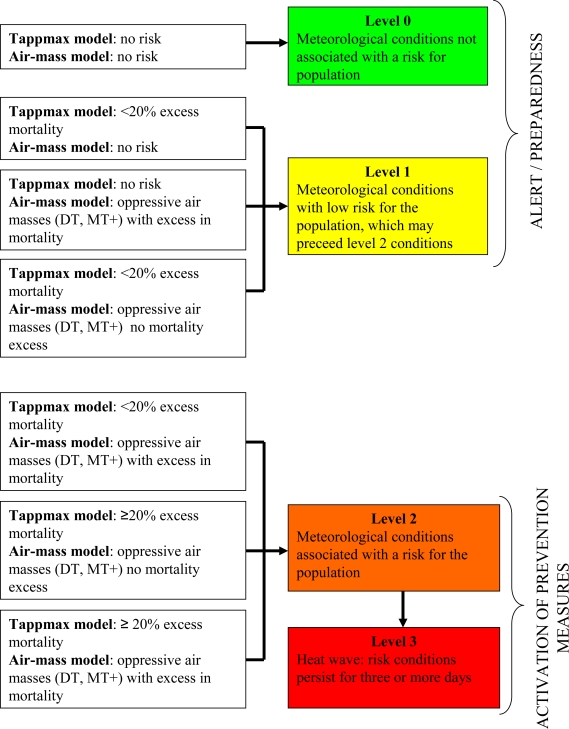
Graded levels of risk in the Italian HHWWS.

During summer, every morning the National Coordination Center receives weather forecast data from the Meteorological Service of the Department of Civil Protection, runs the city-specific models to predict at-risk conditions for the following 72 hours and produces a warning bulletin. The level of risk issued by the HHWWS is graded on the basis of both model outputs as shown in Figure 1. Level 1 (attention) is issued on days with pre-warning meteorological conditions and low risk of mortality; level 2 (alarm) is issued on days with meteorological conditions associated with a high risk for the population, level 3 (emergency) is issued on the third consecutive day of level 2 and identifies heat wave episodes.

At the national level, the warning bulletins for each city are published on the Italian Department of Civil Protection and Ministry of Health websites, while at the local level, the center in charge receives the city-specific bulletin and activates the information network.

## 3. Mortality Surveillance System and Evaluation of the Impact of High Temperature on Mortality

Since 2004, Italy has activated an *ad hoc* rapid mortality surveillance system for the real time monitoring of the impact of heat on mortality during summer [13]. To-date the system is operational all year round in 34 cities. Every day, mortality data for the resident population is sent by local Municipal Registry Offices to the National Coordination Center. Individual records include: date of birth and death, gender, place of death, municipality of birth, residence and death, and the cause of the event (accidental\non-accidental). The dataset can be considered complete on average 72 hours after the day of death is recorded.

The mortality data availability allows for the evaluation of the impact of high temperatures on mortality. Every summer, the effect in the population aged 65 years and over is evaluated using three approaches of increasing methodological and analytical complexity (Table 2):

1. the calculation of excess mortality by month/summer season;
2. the calculation of excess mortality by heat wave episode;
3. the description of the dose-response curve to examine the relationship between Tappmax and mortality.

To estimate the impact of heat on mortality during heat wave episodes and overall throughout summer, excess mortality is calculated as the difference between observed and daily baseline values. Baseline daily mortality is defined as the mean daily value by week and day of the week of the historical time series (Table 2). The analysis of heat wave episodes enables the real-time quantification of the impact of each individual extreme event on mortality. In our system, a heat wave is defined as a period of at least three consecutive days in which HHWWS predicts level 2/3 risk conditions for health (Table 2, Figure 1). Excess mortality is calculated including three days following the heat wave to account for the lag effect of the impact.

The dose-response relationship between mortality and Tappmax is explored in each city using smoothing scatter plots [14] (Table 2). Usually, a typical “J” shape relation is observed during summer with a temperature value corresponding to a minimum mortality (threshold value) above which mortality is increased. Threshold values differ among cities according to the exposure levels to which the local population is typically adapted. To analyze the impact of high temperatures on mortality, the slope of the curve above the threshold is estimated. This analysis also allows identifying possible temporal changes in the temperature-mortality relationship and evaluating the effect of the introduction of heat prevention plans.

**Figure 2.**
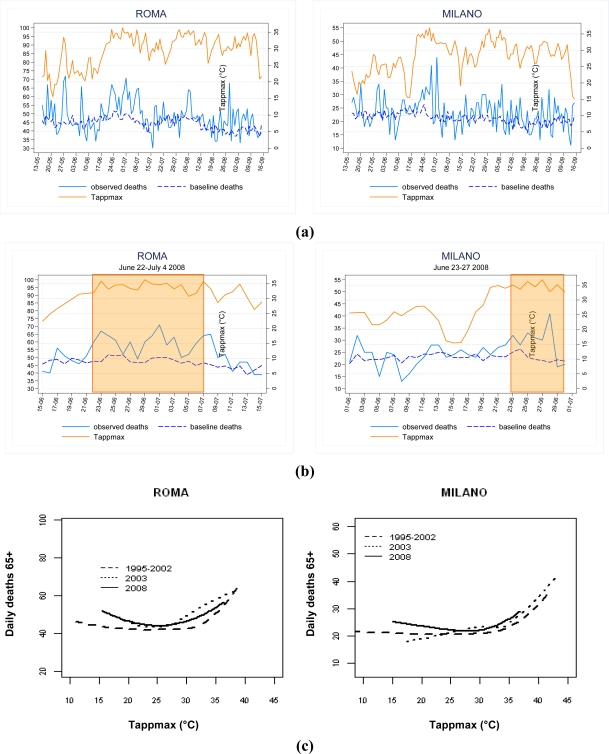
Evaluation of summer mortality. (a) Daily trend of maximum apparent temperature (Tappmax) and observed and baseline mortality in 65 years and over during summer 2008 in two Italian cities; (b) Daily trend of maximum apparent temperature (Tappmax) and observed and baseline mortality in 65 years and over during heat wave episodes in two Italian cities; (c) Relation between maximum apparent temperature (Tappmax) and mortality in 65 years and over during summer in the reference period, 2003 and 2008 in two Italian cities.

As an example, the results of the mortality evaluation carried out during summer 2008 for Rome and Milan are shown in Figure 2. The daily trend in mortality (observed and expected) and maximum apparent temperature values (°C) during summer and during heat wave episodes are reported in Figure 2A and 2B, respectively. In Rome and Milan, the overall excess in mortality for summer 2008 was +8% and 15% (p-value < 0.001), while during the heat wave episodes observed the excess ranged between +22% and +30%, respectively. The dose-response relationship between Tappmax and mortality in the 65+ population in summer 2008, 2003 and during a reference period (1995–2002) shows heterogeneity between time periods and cities (Figure 2C).

## 4. Identification of at-Risk Subgroups

Results of epidemiological studies showed that heat waves have the worse impact on subgroups of the population, including people with impaired physiological and behavioral responses to heat due to their old age [5,15–17], to the presence of chronic illnesses [5,16–18], to limited social contacts [18], to living alone [17], to low socio-economic conditions [5,17], and limited access to air conditioning [18]. The panel of risk factors associated with an increase in mortality may differ among populations due to living conditions, social and cultural context. Three Italian studies have identified a series of socio-demographic factors (living alone, low socio-economic conditions) and health conditions (*i.e.*, respiratory and cardiovascular diseases, metabolic/endocrine disorders, diseases of the central nervous system, depression and psychiatric disorders) which increase the vulnerability to high temperatures [5,17,19]. To our knowledge, Italy is one of the few countries that specifically addresses prevention programs to high risk individuals (susceptible) as recommended by the national guidelines.

In the Italian cities, two procedures are used to identify susceptible individuals depending on the different data sources available. The first method, adopted in 17 cities, uses population registries and data from health information systems. Information on age, gender, civil status and number of family members are obtained from municipal registers while data on median population income for each census block of residence is provided by the Ministry of Finance. In some cities, a small area-based (census tract) socio-economic status indicator using 2001 census data was applied [20]. Information on past hospitalizations (Regional Hospital Discharge Registry) for a group of diagnoses associated with a higher risk of mortality during heat waves are obtained. On the basis of individual characteristics, defined in the literature and/or confirmed through ad hoc studies carried out locally [21], each subject is classified in different risk categories, from low to very high. The proportion of susceptible individuals in the total population aged 65 and over ranges from 0.8% to 5.6% across cities.

The second method, adopted in eight cities, is based on the direct notification by GPs and social workers, taking into account demographic, health characteristics and living conditions. The fraction of susceptible individuals identified is more heterogeneous among cities ranging from 0.4% to 11.6%.

Before summer, the list of subjects aged 65 years and over, inclusive of the individual level of risk, is distributed to health and social services in order to ensure that preventive measures are activated in due time.

## 5. Heat Prevention Plan

In Italy, local prevention plans are based on national guidelines defined by the Ministry of Health. Every year, a survey is performed in all cities to collect information on prevention plans in order to promote a sharing of experiences between local authorities [22]. Table 3 summarizes the main prevention activities and level of implementation, the latter is estimated by considering the number of cities where a specific prevention activity is operational from all the cities participating in the national program.

The Ministry of Health identifies prevention activities to be implemented before the onset of summer, and actions to be activated during the pre-alerting days and during alarm/emergency periods. Each year, an education campaign on the risks of heat is carried out and information on preventive measures is available on the Ministry of Health website. Informative fliers are distributed to centers for the elderly, public places, local pharmacies, health centers and to General Practitioner (GPs). During the summer, a national help-line, managed by medical personnel and trained operators, is activated to provide information on practical measures to reduce health risks during heat waves, on the occurrence of at-risk conditions, and about social and health services available in each city. Furthermore, during heat wave episodes, advice on heat stress avoidance is disseminated via the media.

At the local level, training courses and workshops addressed to GPs, nurses, health care and social workers are organized to raise awareness of risks related to extreme heat waves and to prepare both institutions and personnel to mitigate the impact on health.

A range of social activities are implemented to prevent heat-related effects: a telephone help-line or tele-monitoring, scheduled home visits, and delivery of pharmaceuticals provided by social workers or volunteers. Furthermore, air-conditioned spaces have been implemented in social centers for the elderly and residential care homes and, during heat waves, opening hours are prolonged to provide relief for at-risk individuals.

Health prevention activities involve hospitals, nursing homes, GPs and medical staff. Hospitals and nursing homes define their own emergency protocols, including measures such as postponing non-urgent surgeries, discharge planning during high risk periods, staff rotation restrictions, mobilization of at-risk patients to air-conditioned rooms and increasing bed availability during summer.

In each city, the central component of local prevention plans is the active surveillance of high and very high risk patients by GPs, medical and social personnel. The active surveillance is operated through a dedicated telephone line that triggers a network of health and social services in case of an emergency. In particular, GPs play a central role in actively monitoring patients through specific interventions such as telephone calls and home visits, modulation in pharmacological treatment, home-based treatments, special attention towards at-risk patients discharged from hospital and favoring the access to nursing/residential homes when necessary. When level 2 and level 3 warnings are issued, activities like home visits and treatments are enhanced.

## 6. Discussion

After five years, the Italian heat prevention plan has reached national coverage to include 93% of the population aged over 65 years living in major urban areas. It represents an important example of an integrated approach for the prevention of heat health effects comprising HHWWS, surveillance systems and prevention activities targeted to at-risk population subgroups. The HHWWS system serves as basis for the modulation of prevention measures, and at the local level a good information network is the key component for the efficient running of a heat plan.

Italy was one of the first European countries to develop HHWWS after the exceptional 2003 heat wave [23,24]. More recently, other countries have implemented HHWWS, but they differ greatly with respect to methodology, the information network for warning distribution and the availability of real time outcome data [4]. In most European HHWWS a warning is given in case the forecast temperatures reach or exceed thresholds defined *a priori* and only in some cases based on time series analysis of temperature and mortality [23,24].

In Italy, city-specific HHWWS are based on the retrospective time series analysis of meteorological variables and mortality data providing a reliable epidemiologic basis to account for local climate characteristics and vulnerability of the resident population. This represents a major strength of the Italian HHWWS. Other core elements of the program are the central coordination of HHWWS and a well-structured information network to distribute the warning bulletin to all services involved in local heat-health prevention activities. In some cities, the daily bulletin is used to modulate interventions according to HHWWS risk levels in order to increase efficiency of the whole system.

Following the 2003 heat wave, the interest in the real time evaluation of mortality during summer has increased throughout Europe. Syndromic surveillance and mortality monitoring systems have been developed in some European countries although detailed information on data flow, geographical coverage, type of variables gathered and data collection continuity are not well documented and limited information is available in the literature [25,26]. The availability of real time mortality data during summer is a distinctive feature of the Italian program since it enables the immediate evaluation of the impact of heat waves on mortality; crucial for the timely activation of emergency actions. This data source also allows mortality time series to be updated in order to revise HHWWS models and to provide an indirect evaluation of warning systems and prevention programs. It should be noted that the management of the national mortality surveillance system takes up multiple resources, especially in terms of dedicated personnel, information system support and an efficient collaborative network with local mortality register offices.

The Italian prevention program includes two main components operating at the local level; the social services coordinated by the Municipal offices and Regional Health Agencies, which are the mainstay of the Italian Health Service. Regional Health Agencies have full autonomy in organizational, administrative, financial and technical terms and provide health care services to the population either directly, through their own facilities, or paying for the services provided by independent public and private structures (hospitals and university-managed hospitals). They are also in charge of providing primary care, health education, disease prevention, pharmacies, family planning, child health and information services. A fundamental role is played from GPs, having a central role in primary healthcare delivery. The main activities of GPs include providing medical care, prescribing drugs, ordering diagnostic tests, advising people to specialist services and hospitalizing patients.

The Italian prevention guidelines highlight the importance of targeting prevention activities and available resources to susceptible population subgroups. As documented by the Ministry of Health’s annual survey [22], protocols to identify these subgroups have been defined in several Italian cities. Although the same individual socio-demographic and health characteristics are considered [5,17], the methods applied to define the registries largely differ, due to the availability of data sources, dedicated skilled personnel and financial resources. Therefore, the fraction of susceptible individuals still remains heterogeneous among cities. In most cities data are retrieved from administrative databases to build a population-based registry while in some cities susceptible individuals are directly notified by health (e.g., GPs) and social workers. The latter method is affected by less objective selection criteria adopted by health and social professionals and by differences in the participation rate between cities.

In order to reduce differences in the methodologies for the identification of susceptible subjects, the National Coordination Center recommends the development of local registries based on data retrieved from current administrative databases and defined considering susceptibility factors identified in the population of interest. To the best of our knowledge, the Italian registries of susceptible individuals, although heterogeneous, represent an unique experience in this field, which have no counterpart in other countries. A further explanation of the large difference in the high risk fraction of population identified is also related to the heterogeneity in the availability of health and social services among cities, and is related to specific resources available for the implementation of the summer prevention programs. For example, in cities where interventions are mainly of social assistance a larger proportion of elderly may be involved, whereas in cities where the intervention is provided by the health services a lower proportion of high risk population is identified.

Results from epidemiological studies conducted in Europe and in Italian cities showed absence or low increase of hospitalizations by increasing temperature with no effect of high temperature on hospital admissions for cardiovascular diseases and an effect on respiratory admissions lower than that observed on respiratory mortality [3]. These results suggest that during periods of high temperature many deaths occur rapidly before receiving medical treatment or admission to hospital [27]. These findings suggest that specific preventive measures should be targeted to susceptible individuals residing at home. In some cities GPs carry out an active surveillance on susceptible individuals mostly based on scheduled home visits, modulation in pharmacological treatment and home-based treatments. However, GP participation is on voluntary basis and their involvement is around 30%, entailing a small proportion of susceptible individuals under surveillance [22]. Among the other interventions put in place, informative campaigns and social support activities are well established, while other health actions such as those involving hospitals and nursing homes and training activities have a lower level of implementation. The picture of public health measures that emerges for US and European countries is almost the same [4,28,29]. In general, the critical point is that the potential effectiveness of interventions included in heat prevention plans need still to be formally evaluated.

Evaluation of heat prevention plans as a whole is another crucial issue and despite difficulties, process and outcome assessments should be undertaken [30]. A preliminary evaluation carried out in Italy suggests that a reduction in the impact of heat on mortality has occurred since the introduction of HHWWS and prevention programs [6]. Similar decreasing trends in heat-related mortality have been documented in other countries, although alternative explanations cannot be disregarded [31–34]. There seems to be general consensus that more has to be done in terms of evaluation of HHWWS and prevention measures [4,23].

Another crucial aspect that still requires attention is the timing of activation of HHWWS and prevention activities including the identification of susceptible individuals. In particular the heat plan should be activated sufficiently in advance to be able to cope with heat waves early in the season. Several studies have shown that the highest impact on mortality may occur during the first heat wave episodes [2,6,35,36], when the human organism has not had time to adapt to heat [4]. Furthermore, at the beginning of summer heat waves act on a “full reserve” of susceptible individuals which is gradually depleted by subsequent heat episodes, and consequently, events with similar levels of exposure, may have a lower impact later in the summer [37]. Further efforts are needed to improve the coordination between Italian HHWWS and local prevention activities in order to reduce the impact of heat waves occurring early in the season.

In conclusion, the Italian prevention program is an important example of a collaborative network with a central coordination based on city-specific HHWWS, mortality surveillance system and a wide range of local prevention activities. Improvements are still needed with regards to the coverage of the susceptible populations and the modulation of prevention activities addressed to these subgroups. Moreover, the evaluation of preventive actions is important for the efficient management of resources. After the dramatic media coverage of the 2003 heat wave in Europe, the interest on the effects of climate change on health has suddenly increased. However, in recent years, the attention to this issue has diminished. The preparedness to cope with heat waves becomes a priority in the public health agenda when considering climate change scenarios and the expected increase in frequency and intensity of heat wave episodes in future years.

## Figures and Tables

**Table 1.** Models to predict daily mortality (65 years and over) in the Italian HHWWS.

	**Variables included in the model**	Results*
**Tappmax approach**	*Exposure:* Tappmax= maximum daily value of apparent temperature (Tapp) *T_app_* = −2.653 + 0.994(*T_air_*) + 0.0153(*T_dewpt_*)^2^ *Other variables:* - Holidays - Month (May to August) - Interaction between Tappmax and month - Number of consecutive days with Tappmax above the threshold	**Tappmax thresholds for 10–20% excess mortality:** May: 27.5 °C to 31.5 °C June: 28.5 °C to 34.5 °C July: 29.5 °C to 36.5 °C August: 29.5 °C to 36.5 °C September: 25.5 °C to 35.5 °C **Tappmax thresholds for >20% excess mortality:** May: 28.5 °C to 33.5 °C June: 29.5 °C to 36.5 °C July: 30.5 °C to 39.5 °C August: 30.5 °C to 39.5 °C September: 27.5 °C to 36.5 °C
**Air-mass approach**	For each Air mass:# - Air temperature at 6 a.m.(minimum temperature) - Air temperature at 12 p.m. (maximum temperature) - Time of season (*i.e.*, from 1 for 1st May to 153 for 30th September) - Degree hours (°C)^ - Days in sequence of oppressive air masses (DT, MT+, MT)	**Air mass frequency:** Dry Tropical (DT): 0.4% to 13% Moist Tropical plus (MT+): 1.7% to 15% Moist Tropical (MT): 12.5% to 23% **Excess mortality in 65+ population (%):** Dry Tropical (DT): 7% to 20% Moist Tropical plus (MT+): 15% to 46% Moist Tropical (MT): 4% to 8.6%

* Reported as range between Italian cities

# identified through a Spatial scale synoptic approach [11]

^ Sum of degrees Celsius above 20°C of the four daily temperature values.

**Table 2.** Methods to monitor summer mortality (for the population aged 65 years and over).

**Method**	**Formula**	**Strengths**	**Limitations**
***A) Analysis by month/summer***	Relative excess mortality (%)=(O-E)/E*100 Where: O (observed mortality) = daily mortality of 65+ population E (expected mortality) = baseline daily number of deaths°	Overall summer/month mortality with respect to previous years	Excess depends on the baseline chosen The impact of heat wave episodes not quantifiable Mortality displacement cannot be accounted for
***B) Analysis of heat wave episodes***	Heat wave: ≥ 3 consecutive days with HHWWS level 2 or 3 risk conditions for health plus 3 days following the event Relative excess mortality (%)=(O-E)/E*100 Where: O (observed mortality) = daily mortality of 65+ population E (expected mortality) = baseline daily number of deaths°	Estimation of the impact of extreme exposures Accounts for lag effect of heat on mortality	Excess depends on the baseline chosen Unadjusted for concurrent exposures (*i.e.*, air pollution extremes)
***C) Time-series analysis of relationship between temperature and mortality***	***Shape of exposure-response curve*** Poisson generalized additive model (GAM) with smooth function of *Tapp*max (*i.e.*, penalized regression spline) or locally weighted linear regression	Identifies dose-response relationship Accounts for lag effects Evaluation of geographical and temporal differences in the relationship	Sensitive to outliers Lack of precision when data points are limited

° Calculated as mean daily value by week and day of the week of the historical time series.

**Table 3.** Elements of local heat-prevention plans in Italian cities: summer 2008.

**Preventive measure**	Level of implementation*	**Description**
Written local prevention plan	+++	Guidelines developed at local level including prevention activities and network of health and social services available
***Social interventions***		
Educational campaign	+++	Informative fliers distributed in public places, health centers and General practitioners (GPs). Specific advice disseminated during heat waves.
Telephone help-line	+++	Dedicated help-line providing social support services or regular telephone contact on demand (tele-monitoring)
Social support services	+++	Home visits, personal and home care, and home pharmacy services provided by social workers or volunteers
Availability of air-conditioned places	+	Implementation of air-conditioning units in health and social centers and increase access during heat waves
Educational programmes for social and health workers	++	Training, seminars/workshops, diffusion of specific guidelines among health and social professionals
***Health care interventions***		
Health surveillance of susceptible individuals	++	Phone calls and home visits by GPs. Network of health and social services triggered by a dedicated telephone line
Local register of susceptible individuals	++	Identification of susceptible individuals on the basis of demographic and health characteristics using population registries and health information systems or notification by GPs and social workers
Emergency protocols	++	Emergency measures (*i.e.*, bed redistribution, discharge planning) to improve operational efficiency in hospitals, nursing homes and social structures

* + <50%, ++ 50–75%, +++ 75–100%
